# Qualitative Exploration of Pharmacists’ Feedback Following the Implementation of an “Allergic Rhinitis Clinical Management Pathway (AR-CMaP)” in Australian Community Pharmacies

**DOI:** 10.3390/pharmacy8020090

**Published:** 2020-05-26

**Authors:** Biljana Cvetkovski, Lynn Cheong, Rachel Tan, Vicky Kritikos, Janet Rimmer, Jean Bousquet, Kwok Yan, Sinthia Bosnic-Anticevich

**Affiliations:** 1 Woolcock Institute of Medical Research, The University of Sydney, Camperdown 2050, Australia; rachel.sze.tan@sydney.edu.au (R.T.); vicky.kritikos@sydney.edu.au (V.K.); janetsrimmer@gmail.com (J.R.); sinthia.bosnic-anticevich@sydney.edu.au (S.B.-A.); 2 Discipline of Pharmacy, University of Canberra, Bruce 2617, Australia; Lynn.Cheong@canberra.edu.au; 3 Thoracic Medicine, St Vincent’s Private Hospital, Darlinghurst 2010, Australia; 4 MACVIA-France, Contre les MAladies Chroniques Pour un VIeillissement Actif en France European Innovation Partnership on Active and Healthy Ageing Reference Site, 34000 Montpellier, France; jean.bousquet@orange.fr; 5 Royal Prince Alfred Hospital, Camperdown 2050, Australia; kwokyan@yansydney.com; 6 Sydney Local Health District, Campsie 2194, Sydney, New South Wales, Australia

**Keywords:** Allergic Rhinitis, community pharmacy, management pathway, feedback, interviews, pharmacist, pharmacy intervention

## Abstract

Allergic Rhinitis (AR) is both a common and high burden disease, with the majority of AR sufferers purchasing suboptimal/inappropriate AR medication from community pharmacies. Unfortunately, it is still a challenge to translate the AR management guidelines that are available at both a global and national level into practice. This study aimed to explore the experiences and perceptions of community pharmacists with regards to the implementation of AR management guidelines in real-life everyday practice. This exploration took the form of a qualitative research study in which pharmacists were interviewed following the implementation of a guideline-driven AR management pathway in their pharmacies. Fifteen pharmacists from six pharmacies agreed to participate in a telephone interview. Five themes were identified that encompassed the sentiment of the pharmacists during the interviews: (1) impact of training on pharmacists’ approach to patients and AR management recommendations; (2) patient engagement and the importance of appropriate tools; (3) patient barriers to change in practice; (4) physical, logistical, and inter-professional barriers to change within the pharmacy environment; and (5) recommendations for improvement. The results of this study indicate that, following the implementation of an AR management pathway, pharmacists believe that their interactions with patients around their AR were enhanced through the use of appropriate tools and education. However, if optimal AR management is to be delivered within the community pharmacy setting, the undertaking needs to be collaborative with both pharmacy assistants and general practitioners.

## 1. Introduction

Allergic Rhinitis (AR) is a chronic respiratory condition affecting 19% of Australians, with a prevalence rate in the Australian Capital Territory (ACT) as high as 29% [1]. Poorly controlled AR costs the economy billions of dollars through impairment in quality of life affecting work productivity and school performance and causing absenteeism and sleep disturbances [1,2,3]. The ramifications of poorly controlled AR further extend into co-morbidities, most notably asthma, where it can worsen asthma control and increase the likelihood of asthma flare-ups. 

In Australia, AR can potentially be managed and controlled entirely with pharmacotherapy available to purchase over the counter in community pharmacies without the need to consult a pharmacist. These products are often displayed on shelves which may not be in arm’s reach of the pharmacists but, by regulation, may be in area of the pharmacy which is at a distance from the pharmacist. However, when people with AR do not consult a health care professional (HCP), AR control is rarely achieved [4]. While it has been shown that there is an improvement in AR symptom control when people with AR consult with HCPs and guidelines are followed [5,6], there are several challenges that prevent this from occurring in practice. From the HCP perspective, factors that contribute to the suboptimal control of AR symptoms include the difficulty of translating AR guidelines into practice [7] and an increasing tendency for people with AR to self-select their treatments and bypass HCPs altogether [4].

Research has consistently identified that there is a high proportion of people with AR that self-select their medication, with up to 85% making a suboptimal choice [8] and thus contributing to the worldwide health and socioeconomic burden. [1,2,3,9]. This highlights the urgent need to optimize the role of the pharmacist in the management of AR. Internationally, it is well known and accepted that pharmacists are usually the first line HCP with whom people with AR interact [10]. Pharmacists’ advice is highly influential in the decision-making of a person with AR [11], and including a pharmacist in AR management leads to improved outcomes [12]. While some explorative research has found that the level of pharmacist engagement in AR management is low [4,8], there is no research exploring the reasons behind the low level of pharmacist engagement with AR management in the community pharmacy setting and the failure to fully apply clinical AR guidelines in real-life pharmacy practice.

The implementation of clinical guidelines in healthcare settings is of universal concern and has led to the development of several implementation frameworks, including the “Promoting Action on Research Implementation in Health Services (PARIHS)” framework, designed to ensure the optimal implementation of evidence-based guidelines in healthcare settings [13,14]. The PARIHS framework identifies three key elements—1) evidence, 2) context, and 3) facilitation—that are essential for the successful implementation of guidelines in practice. With regards to AR and the implementation of guidelines, the Allergic Rhinitis Clinical Management Pathway (AR-CMaP) is a guideline pathway for community pharmacists based on this implementation framework [15]. It was specifically developed and implemented to address the physical environment, leadership, culture, and evaluation barriers associated with the implementation of guidelines in practice, as described in PARIHS [13,14].

The aim of this study was to explore the experiences and perceptions of community pharmacists with regards to the implementation of AR management guidelines in real-life everyday practice.

## 2. Materials and Methods 

The ethical aspects of this study have been approved by the Human Research Ethics Committee (HREC) of the University of Sydney (2018/658). It was conducted between October 2019 and November 2019 and all the pharmacist participants signed informed consent prior to enrolment in the study.

### 2.1. Participants

Community pharmacists from the ACT, Australia, were recruited through a list of pharmacies collaborating with the University of Canberra to participate in a research project titled the AR-CMaP project. Their involvement in this project required pharmacists to implement the AR-CMaP in their community pharmacy. AR-CMaP is an AR education and management pathway for community pharmacies designed to support pharmacists in translating AR management guidelines into practice in order to ensure that people with AR in community pharmacies are optimally managed. The AR-CMaP is based on empirical evidence [4,8,16,17,18,19] and the PARIHS framework [13,14]. As part of the implementation process, prior to the delivery of AR care, pharmacists were required to complete the AR-CMaP Education and Implementation Module for pharmacists (a pharmacist needs assessment, followed by an AR-CMaP Webinar with online assessment and then an implementation workshop). The pharmacists were provided with AR-CMaP patient management resources following the workshop, including digital tools (the MASK (Mobile Airways Sentinel Network)-Air mobile application); posters; and patient self-assessment tools, including a Visual Analogue Scale (VAS) Ruler [20] tailored to the implementation needs of each participating pharmacy. The sampling frame for the present study was all pharmacists participating in the AR-CMaP project. Those who agreed to participate and implemented the AR-CMaP in their pharmacies were invited to participate in a telephone interview at the end of the project. While it was accepted that not all pharmacists in each pharmacy would be available to participate in the telephone interview, at least one pharmacist from each pharmacy implementing AR-CMaP was required to participate in order to minimize bias. All the pharmacists signed informed consent prior to participation in the telephone interview.

### 2.2. Semi-Structured Interview Guide 

The semi-structured interview guide (Appendix A) was developed based on empirical evidence on the role of the pharmacist in AR management [4,7,16] and the key principles of context evaluation of PAHRIS framework [13,21]. The interview guide focused on exploring the four key areas of the program implementation: (1) the impact of the educational component on practice; (2) the implementation of the program within the pharmacy environment (physical and cultural); (3) usefulness/challenges associated with the program; and (4) recommendations to improve the program. These four key areas correspond to the exploration of context within the PAHRIS framework, specifically delving into understanding physical, social, cultural, and resource limitations as well as obtaining feedback from individuals to identify weaknesses within the program that need to be addressed to ensure optimal program implementation in the future [21].

### 2.3. Analysis

Data collection and analysis was conducted by members of the research team—RT, BC, LC, and SBA. BC conducted the pharmacist interviews, which were audio recorded and transcribed verbatim by a professional transcription service. RT checked each transcript to ensure transcription fidelity and that any identifying references were removed to ensure all data were de-identified. BC read each interview three times to ensure familiarity prior to the commencement of analysis. BC then conducted line by line observational coding for each interview. The codebook from the observational coding was shared with SBA, RT, and LC for an independent analysis and identification of themes. BC gathered the thematic analysis provided by SBA, RT, LC and BC’s own thematic coding and identified similarities and uniqueness. BC and SBA met to discuss the combined thematic analysis and came to an agreement on five overarching themes that best represented the content of the interviews. These themes were electronically shared with LC and RT, who agreed that these themes represented their individual analysis and the pharmacists’ sentiments.

## 3. Results

In total, 19 community pharmacists implemented the AR-CMaP in six pharmacies and of those, 15 pharmacists (ten female) from six pharmacies were interviewed at the end of the project. All interviews were conducted over the telephone during business hours while the pharmacists were working but able to take a short break. The interview duration varied from 4 min to 14 min (median 10 min). Of the pharmacists who were interviewed, two were proprietors and practicing pharmacists and thirteen were employee practicing pharmacists. There was a minimum of one pharmacist interviewed from each of the pharmacies involved in the implementation of the AR program. The interviews occurred during late October and early November 2019.

The following themes best represent the pharmacists’ responses.

1. Impact of training on pharmacists’ approach to patients and AR management recommendations.

Pharmacists acknowledged they found benefit in receiving more advanced training in AR management. While some reported that the training re-enforced their existing knowledge, most said they welcomed something “new”. In particular, they reported gaining further insights into AR as it related to the high level of burden associated with AR, the skills required to evaluate AR severity, the importance of highlighting the association with asthma to their patients, the broad safety profile of intranasal corticosteroids (INCS), and the steps associated with correct intranasal inhaler technique.

“I’m going for [INCS] first and having more in-depth conversations with people, having conversations about asthma and asthma management as well”

PH#7

As a result of this new knowledge, the pharmacists reported a change in the content, nature, and frequency of their counselling. Pharmacists said they were more proactive in speaking to patients self-selecting AR medications; they included more discussion about intranasal inhaler technique and spoke to their patients with coexisting-asthma about the importance of managing their AR. Counselling often included the evaluation of AR severity while using the VAS ruler. Pharmacists felt more confident in recommending INCS and giving patients better treatment options other than oral antihistamines (OAH). The pharmacists reported positive feedback from their patients following this in-depth counselling.

Some pharmacists used their new knowledge to educate their pharmacy assistants to ensure AR management strategies included all staff within the pharmacy. Educating the pharmacy assistants raised the awareness of the importance of optimal AR management with all the staff, ensuring that people with AR were more likely to get referred to the pharmacist more frequently.

“We always do debrief after we’ve had a training so that we can pass on that information maybe in a slightly simplified version, in a simplified way to them so that they can also use it properly.”

PH#9

2. Patient engagement and the importance of tools.

Pharmacists said that one of the hardest things to do in AR management was to engage patients who chose to self-select and who have been using the same medication for a long period of time. This is where the use of new tools in counselling were important and enabled pharmacists to open up new topics of discussion to engage these patients.

The VAS rulers were particularly well received. Pharmacists reported that the ruler was a useful objective measure that allowed patients to assess the burden/severity of their AR. This provided pharmacists with the opportunity to review the patient’s treatment. Most patients discovered through this process that their AR symptoms were more burdensome than they had realized. Pharmacists reported that this tool made it much easier to convince the patient to consider alternative treatment. However, the difficulty associated with the use of the VAS ruler included keeping a VAS ruler with them all the time so that it was accessible to them when patients were in a rush.

“Being able to show a patient visually [using VAS ruler], if you’re suffering more than a few times a week, then you’re actually needing this treatment in particular, using that format was probably a lot better than what I was doing previously. Yeah, so that definitely helped, and also making sure that patients were a little bit more educated about what it is that’s actually affecting them, rather than just watching the adverts on TV that says, take an oral antihistamine every day. So yeah, I did feel that it has had an impact in a positive way.”

PH#3

Pharmacist’s perspectives on the use of posters elicited mixed responses. Some pharmacists said they initiated conversations about AR between patients and pharmacists, particularly with respect to intranasal inhaler technique. Others said they had no impact because patients were overwhelmed visually and did not take the time to look around their surroundings when selecting a product. Pharmacists also highlighted that they were specifically trying to minimize visual clutter in the pharmacy, especially with large format posters. The posters were useful in some pharmacies, where they prompted patients to ask for the pharmacist and strengthened pharmacists’ counselling message.

“They’ve been quite good. We’ve had some of the posters up on our little promo stand, where we have the allergy stuff, which is really good. A lot of customers are engaging in it and having a good read, so that’s things like the technique of the nasal spray and a bit more about the allergies and that”

PH#1

“The posters and the A4 pages, they don’t work in pharmacy, because… you’ve got very limited space to put things on shelves”

PH#14

The pharmacists reported limited uptake of the MASK-Air app by patients. While they felt that younger patients were more receptive to hearing about it, there was limited download and use of the app overall.

“So the app is a good tool to recommend to patients, however, it doesn’t suit all patients. For example, those who are elderly don’t really know—aren’t that tech savvy—don’t get me wrong, we still told them, but most of them weren’t interested. So depending on what patients you are talking to, depended on whether that was a good tool or not.”

PH#3

3. Patient barriers to change in practice.

Pharmacists regularly cited the patient as a barrier to their ability to improve AR management. Patient beliefs, expectations, and perceptions with respects to their AR were a common concern expressed by pharmacists. While pharmacists appreciated the burden associated with AR and the importance of controlling the symptoms, they felt that patients did not feel the same way and that AR continues to be viewed as a trivial or nuisance condition by the patient. Consequently, patients would frequently rush in and out when visiting the pharmacy to purchase AR medication and appeared to have limited time to speak to the pharmacist. Pharmacists felt that the patients had no expectations of interacting with a pharmacist at that time and did not allow for time to do so. Similarly, pharmacists perceived that patients wanted AR treatments that were convenient and fit seamlessly into their busy lifestyles, such as OAH, preferring them over what pharmacists described as a “dislike” for intranasal medication. Patients’ specific requests for medicines that were sub-optimal were also difficult for the pharmacist to manage. Pharmacists did not want the patient to feel they were disagreeing with them and often used a gentle approach which was not necessarily effective.

“Most of them just trivialise it. So they’re just like, oh, it’s just hay fever. It’s fine, I don’t need to have a conversation about it. So I think that would be the other biggest barrier. So time and the patients just don’t think that it’s a serious sort of condition.”

PH#4

While pharmacists reported that time is a challenge for many patients, the timing of the season and the severity of their symptoms sometimes made them more open to a conversation with the pharmacist. Patients were more willing to listen to what the pharmacist had to say when their symptoms were most severe and could possibly change their mindset/attitude/perception of AR and its management.

“I think, especially now than when we first did the training, not so much but now that we are getting into allergy season and people are having more severe symptoms that they are being more receptive to the nasal sprays”

PH#12

However, pharmacists reported that the cost of prescription medication was a barrier for some patients when purchasing treatments for their AR, specifically prescription INCS sprays, in which case they chose not to have dispensed and asked for a less expensive alternative.

“I think the drawback with both of those is the cost to them. So if people can’t afford that cost then that’s when I would say, oh but we can give you this one it’s not as potent but, it’s worth a go and it’s worth the cost. It costs less.”

PH#12

An additional patient factor that pharmacists reported in AR management was the inability of pharmacists to follow up and monitor their patients. While pharmacists told their patients to come back for a follow up consultation, they very rarely saw the same patient again. Pharmacists in larger pharmacies expected that they may not see the same patient again for follow up but were happy to follow up patients on behalf of other pharmacists. Pharmacists from small pharmacies stated that it was helpful to have regular patients, as it made the follow up of their treatment recommendations a lot easier.

“I always ask them to come back. I don’t always remember if they’re the same person that I recommended something to, but I try and ask them if this doesn’t work in the next few days, please come back and tell me so I can point you in the next direction. Some of our regular customers. Who I do know very well, they do come back. Others? I honestly can’t remember. They might come back. I might not be around.”

PH#9

4. Physical, logistical, and inter-professional barriers to change within the pharmacy environment.

A significant challenge reported by pharmacists was the absence of collaboration and inconsistency in the messages delivered by doctors working within a shared pharmacy and medical center premises. Pharmacists found it difficult to challenge the appropriateness of doctors’ recommendations when they felt it was inappropriate. For example, they reported that many people with AR came from the doctor with a doctor’s note or a prescription for an OAH. Upon review of the patient, pharmacists determined that this was not necessarily an optimal treatment for the patient, however they felt it was not appropriate to challenge the general practitioner’s (GP) recommendations.

“We can’t change the perspective if the doctors are writing script for [OAH], if I say, no you just take intranasal corticosteroid, that’s not good.”

PH#8

The physical layout of the pharmacy also presented a challenge to some pharmacists. Some pharmacies had no comfortable space in between shelves or a private counselling area, and pharmacists felt this limited their ability to deliver effective counselling to their AR patients. Pharmacists with limited free space in their pharmacies also cited the location of the AR medicines as a barrier to the placement of posters and access to VAS rulers when needed.

“We found it a bit tricky to actually get the [posters] directly at the allergy section within the pharmacy, only because of where it’s actually situated in the pharmacy.”

PH#5

“The hay fever section needs to be easily accessible for the pharmacists. The pharmacist needs to be having conversations in that space. I think that the—and obviously, if you’re in the dispensary, you need to have—it needs to be within eye-view of that section at least”

PH#7

While the AR-CMaP program expected all pharmacists within a pharmacy to have participated, some pharmacies experienced logistical challenges and were not able to enroll all pharmacists in the program. These pharmacies subsequently experienced difficulties in ensuring a consistent message was being delivered within the pharmacy about AR management.

“I was the only one who actually did the official training, in the webinar, and I just did a condensed version—like a verbal condensed version—to the other pharmacists that work here. I did send them the material to do but they didn’t end up doing it because it was… …fairly time-consuming. So I’d say if you could condense that, that would help”

PH#14

5. Recommendations for improvement.

Many pharmacists felt that the implementation of AR management guidelines into real-life everyday practice should be collaborative, with the AR-CMaP program broadened to include pharmacy assistants and GPs. Most pharmacists tried to share the main AR management learnings with pharmacy staff (including pharmacy assistants) during staff meetings. However, they felt strongly that if pharmacy assistants attended the same training as the pharmacists or were provided specific pharmacy assistant education, it would have been beneficial. Similarly, the pharmacists stated that if their neighboring GP colleagues were provided with the same training at the same time, it would ensure a consistent message was delivered to their common patient community and would re-enforce the recommended AR management pathways.

“We probably didn’t train them [pharmacy assistants] as well as we could’ve”

PH#3

“I spoke to the [pharmacy assistants] after I had done the training, and I filled them in on what the training was, what the guidelines and treatments were for the different levels of various symptoms, et cetera. They were interested to know about that because they didn’t necessarily know that that was the right thing. So they were quite surprised to find out about the oral antihistamines, and how they’re fairly ineffective for most”

PH#14

Improvements to patient tools and resources were also suggested. Pharmacists felt that paper resources such as posters were outdated and were not modern forms of communication. Pharmacists recommended adopting technology and social media to raise awareness of AR and its burden. Pharmacists believed that by employing social media to engage patients, they would be more receptive to the information, rather than when they were in a rush in the pharmacy. Social media was recommended as a platform to raise awareness of the importance of speaking to a pharmacist about AR. Pharmacists also recommended that technology be used in an interactive way in the pharmacy. Suggestions included the digitalization of the VAS ruler and its placement near AR medicines to encourage patient interaction. It was felt that by using an interactive tool, patients would be motivated to speak to the pharmacist about their AR treatment. Alternatively, pharmacists suggested reducing the size of the posters and VAS ruler so that they can be attached to the pharmacy shelves to grab the attention of the patients.

“[The patient will] be sitting at home after dinner, or in front of the TV with their phone skimming through stuff and they’ll see an interesting post on allergic rhinitis and think, oh, that sounds like what I’ve got. Read into it and then find, through their own investigation, maybe there’s a better treatment available”

PH#2

Pharmacists suggested that in order to maximize participation and minimize logistical issues, a shorter pharmacist education component would have been beneficial. Although they understood extensive education was required to meet Continuing Professional Development (CPD) requirements, they felt a more concise program would be more impactful as it would be accessed by more pharmacists. The length of the webinar also meant that in some circumstances, it had to be completed outside of working hours. In some circumstances, this resulted in pharmacists being paid by their employers to complete the training after hours.

“The webinar one was interesting. It was probably a little bit too long. I understand it probably needs all those bits in there. But that was a little bit long.”

PH#12

Pharmacists reported that the timing of the delivery of the implementation program was a factor that required improvement. The pharmacists felt that they were not given enough time to mentally and physically prepare for the implementation prior to the peak of AR season. Preparations would ideally have begun approximately three months prior to spring to ensure enough time for all staff to be engaged and physical changes to the pharmacy environment to be made.

“I think there were some timelines and deadlines that had to be met, coming up to the allergy season, so I think it will kind of add constraints there but yeah probably just maybe starting maybe a month earlier, get in the training—the outline of the training and what needs to be done, just to get everyone prepared”

PH#5

## 4. Discussion

This study, to our knowledge, is the first to explore pharmacists’ feedback following the implementation of an AR clinical management pathway designed to translate AR management guidelines into practice and optimize AR management within the community pharmacy setting. Several key areas for consideration were identified, however it was clear that optimal AR management requires a multi-pronged approach which goes beyond the pharmacist. It was evident from the research that AR management must be a collaborative effort between pharmacists, GPs, pharmacy assistants, and their patients, and pharmacists must be equipped with comprehensive tools and strategies to communicate with and engage patients about optimizing their AR treatment.

While the community pharmacy is central to AR management and pharmacists clearly are able to improve AR outcomes, very few pharmacist/pharmacy-centered AR interventions have been explored [22,23,24]. AR management guidelines developed by the Allergic Rhinitis and its Impact on Asthma (ARIA) organization recognize the importance of the pharmacist’s role in AR management, however the dissemination and use of the guidelines is limited [25,26]. It may be perceived that pharmacists know everything there is to know about AR management, but even those who felt they were more knowledgeable valued and benefited from more advanced knowledge acquisition, demonstrating that AR is not a simple condition with simple management. This study suggested that they needed this advanced knowledge to result in practice change, noting confidence as one of the key factors, especially in the context of high patient self-management behaviors.

Pharmacists embraced the new knowledge they had gained to engage with patients that would otherwise have chosen not to interact with a pharmacist. The pharmacists welcomed the tools provided such as the MASK-air app, VAS ruler, and AR posters as items that also sparked opportunity for new conversations with patients that would otherwise have been missed. This study also suggests that pharmacists are not accustomed to having conversations with patients around AR severity or the disease itself, and any tool or objective measure of AR severity is desperately needed within the pharmacy setting. A further reason why pharmacists may not be used to having conversations with patients around their AR is that they may perceive that their patients may not need to speak to them. A recent study found that patients feel confident in self-managing their AR and they do not see the need to speak to the pharmacist [17], propagating the pharmacists reduced confidence in communication.

Pharmacists believed that addressing the issue of AR management needed to be a whole pharmacy approach including pharmacy assistants, as well as a collaborative approach across the health care system. Pharmacists reported difficulty when inappropriate AR treatments were being recommended by neighboring GPs to patients. To improve AR management within the community pharmacy setting, the pharmacists recommended collaborative AR training with GPs to ensure a consistent message is delivered to AR sufferers that is consistent with ARIA guidelines, incorporating these values into an integrated care approach [7]. Although in some non-Australian environments the GP is central to the management of AR [25], in Australia and countries with similar health care systems where there is an extensive availability of AR treatments in pharmacy and limited access to GP appointments [27], it is essential that pharmacists and GPs work collaboratively to manage AR and support their patients with AR self-management. 

The pharmacy environment, technology, and support tools were highlighted as areas requiring improvement; however, it appears that a systemic change may be required to improve AR management in the community pharmacy. The location of the medications was clearly a barrier—i.e., the location was highly important when it came to the opportunity to counsel patients about their AR. Therefore, perhaps AR medications, despite being available for purchase without a prescription and without interaction with the pharmacist, should be located in a position which makes them difficult to access without pharmacist interaction.

The principles of AR management also need to promote the follow up and review of AR patients. Other chronic respiratory conditions such as asthma and chronic obstructive pulmonaru disease (COPD)have systems in place to support patient self-management and follow up with a HCP [28], which is lacking in AR [29]. Pharmacists will continue to experience difficulty monitoring their AR patients if they are not supported with universal change to the principles of AR management [18]. Pharmacists need to also be equipped with tools with which to objectively measure a patient’s AR severity and support the monitoring of medication effectiveness. The inability of HCPs to objectively measure AR severity has been highlighted in literature previously [30], and the impact of this on optimising AR management in primary care has further been confirmed by this study.

Although this study is limited by its small sample size, it is valuable in its methodology and the unique location chosen for its high AR prevalence. AR pathways are traditionally evaluated by the changes in clinical outcomes that they generate [31]. However, this study employed the principles of evaluation, where the interest was in finding out what worked well, what did not, why, and in what context, in order to further inform the development of the program [31], as well as exploring physical, social, cultural, and resource limitations from the perspective of individuals within the PARIHS implementation framework [21].

## 5. Conclusions

This study demonstrates that AR management within the community pharmacy is complex and several barriers need to be addressed in order to implement a clinical AR management pathway for pharmacists. To optimize AR management, a collaborative approach must be adopted within the primary care setting, and pharmacists need to be provided with resources to support self-management among their patients with AR.

## Appendix A. Interview Guide to Evaluate the Intervention of AR-CMaP

**Table A1:** Interview Guide.

Topics	Primary Question	Secondary Question	Clarification
Education	What is the most important (three key) things you learnt about the training/through the process?	Has your perspective of AR management changed and how?	What things, if any, have you done differently for AR management in your pharmacy?
Implementation	What is overall feedback on the implementation of AR-CMaP in your pharmacy?	What strategies did you implement and why/why not?	Who was responsible for implementing these strategies? Pharmacists? Assistants? Why was this the case? What did others within your pharmacy think about the process?
Usefulness/challenges of implementation	What elements were most useful? Why?/why not?	How did you believe they were most useful? What were the challenges you experienced in their usage?	What did the other pharmacy staff think of the implementation and whether it was useful?
Recommendations	What recommendations do you have to improve AR management in the pharmacy in the near future?	How do you feel about the different aspects of AR management in pharmacy? Pharmacist training/range and effectiveness of products/ease of access to information and guidelines/patient engagement.	What are the hardest things about optimizing AR management within the pharmacy setting? What things do you think work well in AR management in pharmacy?
